# Effect of Race and Tumor Subsite on Survival Outcome in Early- and Late-Onset Colorectal Cancer

**DOI:** 10.3390/cancers18020180

**Published:** 2026-01-06

**Authors:** Mei-Chin Hsieh, Elena M. Stoffel, Kristen Purrington, Xiao-Cheng Wu, Jaeil Ahn, Siddhi Patil, Shengdi Wen, Muhammed Jawla, Batsirai Mabvakure, Laura S. Rozek

**Affiliations:** 1 Epidemiology and Population Health Program, Louisiana Tumor Registry, School of Public Health, Louisiana State University Health-New Orleans, New Orleans, LA 70112, USA; xwu@lsuhsc.edu (X.-C.W.); swen@lsuhsc.edu (S.W.); mjawla@lsuhsc.edu (M.J.); 2 Division of Gastroenterology, University of Michigan Medical School, Ann Arbor, MI 48109, USA; estoffel@med.umich.edu; 3 School of Medicine, Wayne State University, Detroit, MI 48202, USA; fn6868@wayne.edu; 4 Department of Biostatistics, Bioinformatics, and Biomathematics, Georgetown University Medical Center, Georgetown University, Washington, DC 20057, USA; ja1030@georgetown.edu; 5 Georgetown-Lombardi Comprehensive Cancer Center, Georgetown University, Washington, DC 20057, USA; sp1602@georgetown.edu (S.P.); bm1173@georgetown.edu (B.M.); lr898@georgetown.edu (L.S.R.)

**Keywords:** colorectal cancer, early-onset CRC, late-onset CRC, survival, racial disparities, anatomic subsite disparities

## Abstract

**Background**: While colorectal cancer (CRC) incidence and mortality have declined among patients aged ≥50 years (late-onset), rates are increasing in those aged <50 years (early-onset). Historically, non-Hispanic Whites (NHW) have had better 5-year survival compared with non-Hispanic Blacks (NHB), and rectal cancer has had better outcomes than colon cancer. Whether these disparities by race and tumor location are evident for both early-onset (EOCRC) and late-onset (LOCRC) CRC remains unclear. **Methods**: CRC cases diagnosed from 2011 to 2022 were identified from the Louisiana Tumor Registry. EOCRC was defined as diagnoses at ages 20–49 years, and LOCRC was defined as diagnoses at ages ≥50 years. Racial groups included NHW and NHB; tumor location was categorized as proximal colon, distal colon, or rectum. Cox regression was used to assess unadjusted and adjusted overall and cancer-specific survival. **Results**: Of 23,738 CRC patients, 10.7% were diagnosed at age <50 years. Compared to LOCRC, EOCRC patients included a higher proportion of NHB (37.5% vs. 32.6%) and rectal tumors (44.4% vs. 29.9%). NHB had worse overall survival than NHW in early-onset distal colon cancer (adjusted HR [aHR] = 1.358; 95%CI: 1.024–1.801). Conversely, NHB had better overall (aHR = 0.899; 95%CI: 0.831–0.973) and cancer-specific survival (aHR = 0.873; 95%CI: 0.793–0.960) in late-onset rectal cancer. Among EOCRC NHW, proximal tumors were associated with worse overall (aHR = 1.407; 95%CI: 1.102–1.796) and cancer-specific survival (aHR = 1.379; 95%CI: 1.057–1.799) compared with distal tumors. **Conclusions:** Survival differences by race and tumor subsite are observed between EOCRC and LOCRC, with NHB showing a lower hazard of death in some LOCRC subgroups. These findings highlight the need to consider the age of onset and tumor location when addressing racial disparities in CRC outcomes.

## Simple Summary

The aim of this retrospective cohort study was to examine the effects of race and anatomic subsites on survival in early-onset (EOCRC) and late-onset colorectal cancer (LOCRC). In Louisiana, 10.7% of CRC cases were diagnosed at ages 20–49 years and were more likely to be non-Hispanic Blacks (NHB), female, rectal, and diagnosed at a distant stage compared with LOCRC. Overall racial disparities in survival were attenuated after full adjustment in both age groups. However, subsite-stratified analyses revealed heterogeneity. Among EOCRC patients with distal colon or rectal cancer, NHB had worse cancer-specific survival than non-Hispanic Whites (NHW). In contrast, among LOCRC patients with rectal cancer, NHB showed better survival than NHW. Survival patterns by anatomic subsite varied by race and age, with worse survival for early-onset proximal colon cancer among NHW and better survival for late-onset rectal cancer among NHB only.

## 1. Introduction

Colorectal cancer (CRC) ranks as the third most commonly diagnosed cancer and the second leading cause of cancer-related deaths in the United States, with an estimated 154,270 new cases and 52,900 deaths projected in 2025 [1]. National and Louisiana trends over the past three decades have shown a steady decline in CRC incidence and mortality, a pattern largely attributed to changes in risk behaviors as well as improvements in screening practices and treatment modalities [1,2,3,4]. Despite this progress, Louisiana continues to experience CRC incidence and mortality rates that exceed the national average [5].

While CRC incidence increases with age, with rates rising progressively in older populations, a concerning global trend has emerged: CRC diagnoses are increasing among younger adults, particularly in high-income countries [6,7,8]. In the United States, where CRC screening is widely accessible, there has been a notable decline in both the incidence and mortality of CRC among patients aged 50 years and older, especially those aged 65 years and above, who experienced an average annual decrease of 3.4% in incidence from 2010 to 2019 and a 2.9% decrease in mortality from 2011 to 2020 [4]. In contrast, the opposite trend is observed in younger populations. Among patients under the age of 50 years, the incidence of CRC has increased by 1.7% annually, and mortality has risen by 1.2% [4]. Although the causes of early-onset CRC (EOCRC) are not clear, it is unlikely to be due solely to familial risk, as approximately one in five EOCRC carries a germline mutation [9]. This points to cohort-specific shared exposures, such as diet (specifically ultra-processed foods), emerging chemicals, antibiotic use, and sedentary lifestyles, all of which may contribute to chronic inflammation and increased susceptibility to EOCRC [10]. The relationships between race, social factors, and environmental burden are complex. High social and environmental burden has been associated with EOCRC mortality in NHB (but not NHW) [11], indicating that environmental and social factors likely influence the distribution of and survival from EOCRC, especially in racially and economically diverse areas of the U.S.

Overall, individuals with EOCRC tend to have better survival compared with late-onset CRC (LOCRC), despite being diagnosed at later stages, as LOCRC is often diagnosed at screening [4,12,13]. Both increasing number of comorbidities and age adversely influence CRC prognosis [14]. Despite this, persistent racial disparities in CRC outcomes remain evident. Non-Hispanic Blacks (NHB) consistently experience worse survival rates than non-Hispanic Whites (NHW), regardless of age group or stage at diagnosis [1,2,4,13,15,16,17,18,19]. Nationally, between 2014 and 2020, the 5-year related survival rate for CRC was 65% among NHW compared to 59% among NHB [17], with similar disparities observed in Louisiana [5]. Anatomic subsite also influences outcomes. Among adults, those diagnosed with right-sided (proximal) colon cancers exhibit a higher risk of mortality compared with those who have left-sided (distal) colon cancers [20,21,22,23]. Furthermore, rectal cancer is associated with a higher 5-year relative survival rate than colon cancer (67% vs. 63%) and is more common among younger patients [1,24].

Most prior studies assessing racial and subsite disparities in CRC survival either combined all age groups or did not control for certain variables that may impact survival, such as patients’ existing comorbid conditions and/or treatment status. It remains uncertain whether these disparities in survival persist in EOCRC and LOCRC after adjusting for potential confounders and whether effect modification exists between race and tumor location. This study aimed to (1) compare survival outcomes between EOCRC and LOCRC after adjusting for potential confounders; (2) assess the influence of race and anatomic subsite on survival in EOCRC and LOCRC; and (3) evaluate the effect modification of race and anatomic subsite on survival outcomes in both EOCRC and LOCRC. We hypothesized that the association between race and survival differs by tumor subsite and age of onset and that these interactions persist after adjustment for sociodemographic, clinical, and treatment-related factors. Our analysis takes advantage of data collected by the Louisiana Tumor Registry, whose catchment area of the state of Louisiana is racially diverse with unique environmental exposures [25].

## 2. Materials and Methods

### 2.1. Data Source and Study Cohort

Data on colorectal cancer (CRC), classified using the International Classification of Diseases for Oncology, Third Edition (ICD-O-3), were obtained from the Louisiana Tumor Registry (LTR) to conduct a retrospective population-based cohort study. The LTR, a statewide population-based cancer registry, is supported by the Centers for Disease Control and Prevention (CDC)’s National Program of Cancer Registries (NPCR) and the National Cancer Institute (NCI)’s Surveillance, Epidemiology, and End Results (SEER) program to collect high-quality cancer data, including cancer staging, treatment, and survival data.

The eligibility criteria included invasive CRC cases diagnosed between 2011 and 2022 in individuals aged 20 years and older. We restricted the cohort to patients coded as White or Black due to the small number of individuals from other racial groups in Louisiana. ICD-O-3 histology coded to 9050–9055, 9140, or 9590–9993 or anatomic sites coded to appendiceal site (C181), overlapping lesion of colon (C188), or colon not otherwise specified (C189) were excluded. CRC cases identified through autopsy reports or death certificates as well as patients who died on the same date as the CRC diagnosis were excluded.

### 2.2. Sociodemographic and Clinical Variables

EOCRC was defined as CRC diagnosed between the ages of 20 and 49 years, while LOCRC was defined as CRC diagnosed at age 50 years or older. Racial categories included non-Hispanic Whites (NHW) and non-Hispanic Blacks (NHB). Other sociodemographic variables included sex, marital status (married including domestic partner, single including unmarried/separated/widowed/divorced, and unknown), type of insurance at the time of cancer diagnosis and/or treatment (private insurance, Medicare/other government, Medicaid, and uninsured/unknown), census tract level poverty, and urban/rural status. The poverty rate at the census tract level for the diagnosis address was the population percentage below the official poverty threshold, according to the American Community Survey, and was categorized into three groups: <10%, 10%–<20%, and ≥20%. Urban/rural status was based on the 2010 Rural Urban Commuting Area (RUCA) tract level, which classified census tracts into urban or rural by using measures of population density, urbanization, and daily commuting.

Anatomic subsite of CRC was categorized as proximal colon including cecum (C180), ascending (C182), hepatic flexure (C183), and transverse colon (C184); distal colon including splenic flexure (C185), descending (C186), and sigmoid colon (C187); and rectum including rectosigmoid junction (C199) and rectum (C209). Other clinical variables included cancer stage, histology grade, tumor number (single primary or more than one primary), comorbid conditions, and the first-course treatment. The cancer stages (localized, regional, and distant) were collected using SEER summary stage 2000 for cases diagnosed in 2011–2017 and SEER summary stage 2018 for cases diagnosed in 2018–2022 [26,27]. The histology grade was categorized into low (well and moderately differentiated combined), high (poorly differentiated and undifferentiated grade combined), and unknown grades. Comorbid conditions, as coded in the medical records using either ICD-9-CM or ICD-10-CM codes, were converted into the Charlson comorbidity index (CCI) score = 1, CCI score ≥ 2, and no comorbidity documented in the medical records based on Deyo’s enhanced CCI score [28]. The first-course treatment, including surgery, chemotherapy, and radiation therapy, was categorized into no, yes, and unknown for each type of treatment received.

### 2.3. Survival Endpoints

This study assessed overall and CRC cause-specific survival. Eligible patients were followed up to 31 December 2023, if alive. The survival duration was the time between the date of initial diagnosis and the date of death, the date of last contact, or the date of closing follow-up, if alive. The underlying cause of death for deceased patients was obtained from the state or the national death file. The event for overall survival was death from any cause. The definition of cancer-specific survival was based on the SEER ICD-10 cause-specific death classification [29]. We grouped cancer-specific outcome into three categories: alive, CRC-related death, and death from other causes; individuals who were alive or died of other causes were classified as censored at last follow-up.

### 2.4. Statistical Analysis

Descriptive statistics were used to summarize patient sociodemographic characteristics, clinical factors, treatment modalities, and vital status across age groups. Pearson’s chi-square test was employed to evaluate bivariate associations between categorical variables. The Kaplan–Meier (KM) method and log-rank tests were used to compare the KM curves by sociodemographic and anatomic subsite. We used the Cox proportional hazards regression model to examine the association between the primary explanatory variables of interest (age group, race, and anatomic subsite) and survival for both crude and adjusted models. To assess associations with cancer-specific survival, we employed a cause-specific hazards model to account for competing risks. This approach is commonly used in cancer survival studies, particularly when competing events such as death from other causes are relatively uncommon among cancer patients. Effect modification was assessed by including an interaction term between race and anatomic subsite in the fully adjusted Cox model. The proportional hazards assumption for the Cox models was evaluated using Schoenfeld residuals. All statistical analyses were performed using SAS version 9.4 (SAS Institute Inc., Cary, NC, USA). Statistical significance was assessed using two-sided tests with a significance level set at 0.05.

## 3. Results

This study included 23,738 individuals diagnosed with CRC during the 12-year period, and 10.7% were diagnosed with CRC at age <50 years (EOCRC). The EOCRC group contained a higher proportion of NHB and females compared to the late-onset CRC (LOCRC) group: 37.5% vs. 32.6% and 48.6% vs. 45.7%, respectively (Table 1). EOCRC individuals were more likely to be diagnosed with rectal cancer (44.4%) and at a distant stage (26.9%), while LOCRC cases were more likely to be diagnosed with proximal colon cancer (43.1%) and localized stage (41.3%). Overall, EOCRC had a better 5-year survival rate than LOCRC in both all-cause death (65.1% vs. 52.7%, *p* < 0.0001) and cancer-specific death (68.5% vs. 65.5%, *p* < 0.0001) (Table 1).

**Figure 1:**
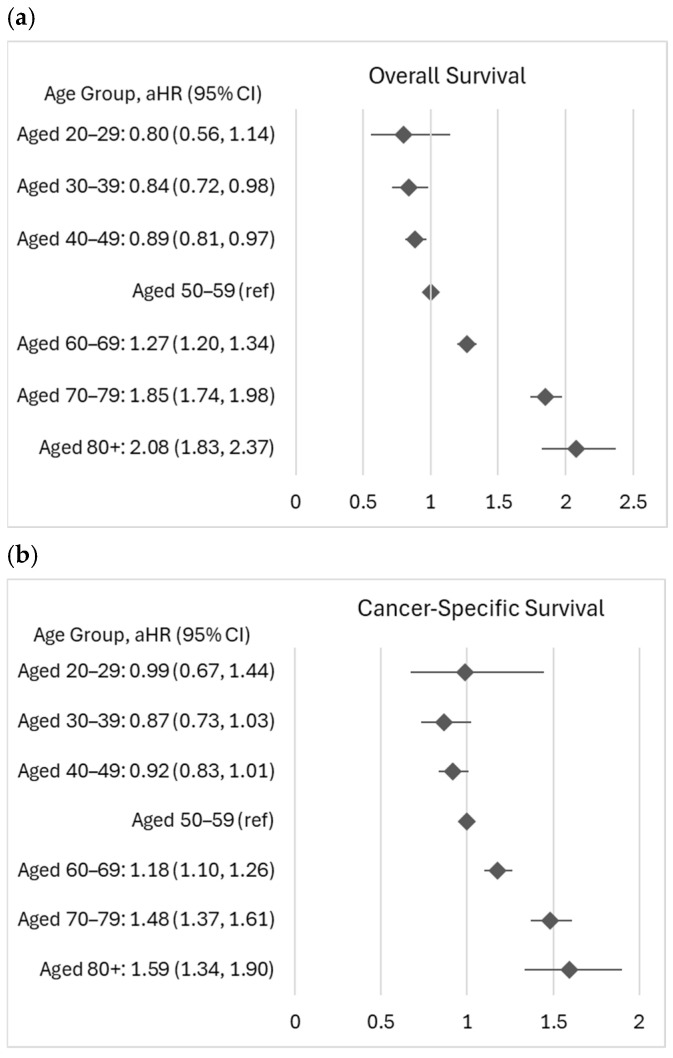
Adjusted hazard ratios (aHRs) ^1^ by 10-year increment of age. (**a**) Overall survival; (**b**) cancer-specific survival; ^1^ adjusted for race, sex, marital, insurance, census tract level poverty, urban, stage, grade, number of tumors, comorbidity, and treatment. Abbreviation: CI, confidence interval.

Figure 1 shows the adjusted hazard ratios (aHRs) by a 10-year increment of age at diagnosis. Using age 50–59 years as a reference, we found that aHR increased as age increased for both all-cause and cancer-specific death, except for those aged 20–29 years in cancer-specific death. For all-cause death, CRC patients aged 30–39 years and 40–49 years had better survival than those aged 50–59 years after controlling for demographic, clinical, and treatment factors, with aHR 0.84 (95%CI: 0.72–0.98) and 0.98 (95%CI: 0.81–0.97), respectively. Although there were no significant differences in cancer-specific survival among patients aged <50 years and those aged 50–59 years, the increasing aHRs were similar with overall survival, except age 20–29 years.

### 3.1. Racial Differences in Survival

**Figure 2:**
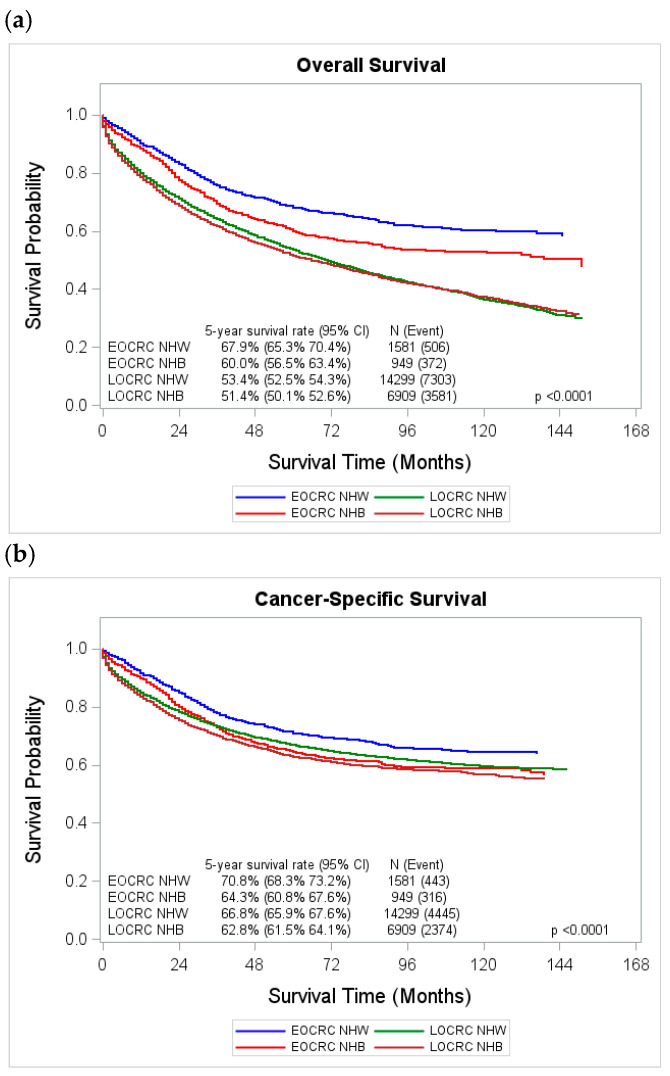
Unadjusted overall survival and cancer-specific survival curves by race and age group, Louisiana 2011–2022. (**a**) Overall survival by race and age group; (**b**) cancer-specific survival by race and age group. Abbreviations: EOCRC, early-onset colorectal cancer; LOCRC, late-onset colorectal cancer; NHW, non-Hispanic Whites; NHB, non-Hispanic Blacks.

Figure 2 presents the unadjusted Kaplan–Meier survival curves by race and age group. Among patients with EOCRC, NHW had significantly better overall survival compared to NHB (*p* < 0.0001). The 5-year overall survival rate was 67.9% (95%CI: 65.3–70.4%) for NHW and 60.0% (95%CI: 56.5–63.4%) for NHB (*p* = 0.0013) (Figure 2a). In contrast, among patients with LOCRC, overall survival did not differ significantly between the two racial groups (*p* = 0.0860). The 5-year overall survival rates were similar, 53.4% (95%CI: 52.5–54.3%) for NHW and 51.4% (95%CI: 50.1–52.6%) for NHB (*p* = 0.3098). For cancer-specific survival, NHW demonstrated significantly better survival than NHB in both age groups (Figure 2b). Among EOCRC patients, the 5-year cancer-specific survival rate was 70.8% (95%CI: 68.3–73.2%) for NHW and 64.3% (95%CI: 60.8–67.6%) for NHB (*p* = 0.0001). In LOCRC, the 5-year cancer-specific survival rate was 66.8% (95%CI: 65.9–67.6%) for NHW compared with 62.8% (95%CI: 61.5–64.1%) for NHB (*p* < 0.0001).

Unadjusted Cox proportional hazards regression results showed NHB with EOCRC had a 30.7% higher hazard of all-cause death (HR = 1.307; 95%CI: 1.143–1.494) and a 26.9% higher hazard of cancer-specific death than NHW (HR = 1.269; 95%CI: 1.099–1.466) (Table 2). Among patients with LOCRC, an unadjusted racial disparity was observed only in cancer-specific survival, where NHB had a 12.7% higher hazard of cancer-specific death compared to NHW (HR = 1.127; 95%CI: 1.072–1.184). However, after adjusting for all covariates, these racial disparities were no longer statistically significant for either age group on both survival outcomes (Table 2). We further examined whether the effect of race on survival varied across different anatomic subsites. Among EOCRC, a significant racial disparity was observed in cancer-specific survival for those diagnosed with distal colon cancer; NHB had a 35.8% higher hazard of cancer-specific death (aHR = 1.358; 95%CI: 1.024–1.801) compared to NHW. Among LOCRC patients, racial differences were observed for those diagnosed with rectal cancer. NHB demonstrated better survival compared to NHW for both overall and cancer-specific survival, with aHR of 0.899 (95%CI: 0.831–0.973) and 0.873 (95%CI: 0.793–0.960), respectively (Table 2).

### 3.2. Anatomic Subsite Differences in Survival

**Figure 3:**
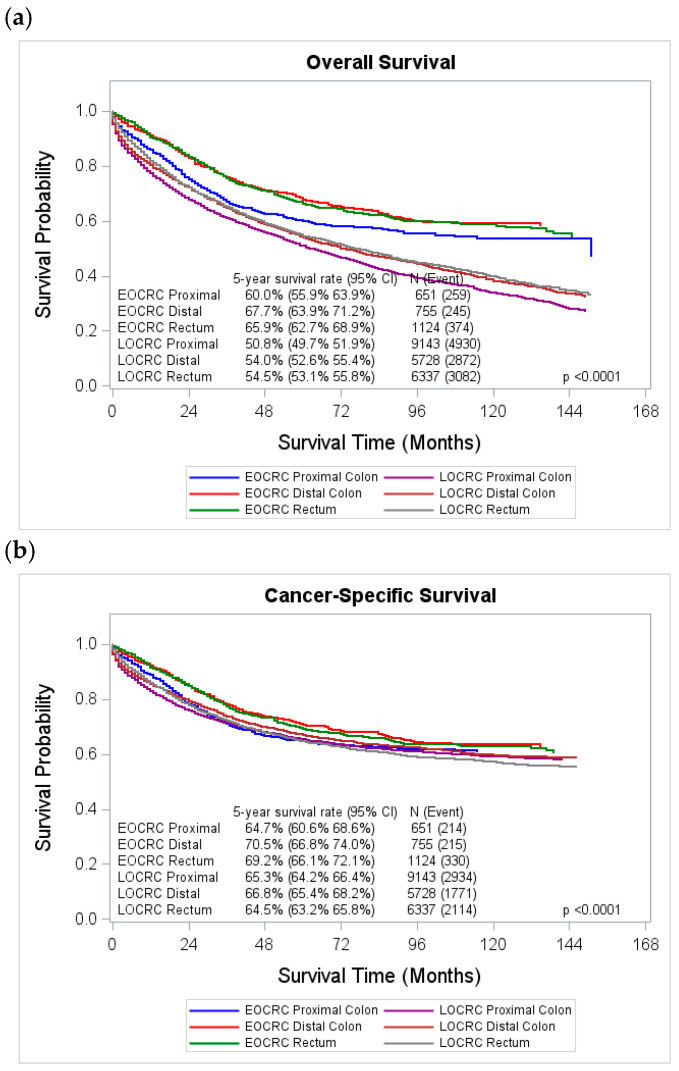
Unadjusted overall survival and cancer-specific survival curves by anatomic subsite and age group, Louisiana 2011–2022. (**a**) Overall survival by subsite and age group; (**b**) cancer-specific survival by subsite and age group. Abbreviations: EOCRC, early-onset colorectal cancer; LOCRC, late-onset colorectal cancer.

When examining survival differences by tumor subsite, we found that patients diagnosed with distal colon or rectal cancer had similar unadjusted overall survival, both of which were higher than for those diagnosed with proximal colon cancer, across both age groups. Five-year overall survival ranged from 67.7% (95%CI: 63.9–71.2%) for early-onset distal colon cancer to 50.8% (95%CI: 49.7–51.9%) for late-onset proximal colon cancer (Figure 3a). While cancer-specific survival followed a similar pattern to overall survival for both EOCRC and LOCRC, a notable exception was observed for proximal colon cancer: early-onset cases had a slightly lower 5-year cancer-specific survival rate (64.7%, 95%CI: 60.6–68.6%) compared to late-onset cases (65.3%, 95%CI: 64.2–66.4%) (Figure 3b).

Compared with patients who had distal colon cancer, those with proximal colon cancer showed a higher hazard of death for both survival outcomes across EOCRC and LOCRC in the unadjusted Cox models (Table 2). However, among patients with rectal cancer, only those with LOCRC exhibited a slightly higher hazard of cancer-specific death (HR = 1.071; 95%CI: 1.005–1.140). After adjusting for all covariates, subsite differences in survival remained statistically significant solely for all-cause death. Compared to distal colon cancer, EOCRC patients with proximal colon tumors had a 22.5% higher hazard of all-cause death (aHR = 1.225; 95%CI: 1.026–1.462), while LOCRC patients with rectal cancer showed slightly better overall survival (aHR = 0.910; 95%CI: 0.859–0.964) (Table 2). After stratifying by race, subsite disparities emerged among NHW with EOCRC and among NHB with LOCRC. Among NHW with EOCRC, proximal colon cancer was associated with higher mortality, including a 40.7% higher hazard of all-cause death (aHR = 1.407; 95%CI: 1.102–1.796) and a 37.9% higher hazard of cancer-specific death (aHR = 1.379; 95%CI: 1.057–1.799) compared with distal colon cancer. In contrast, among NHB with LOCRC, rectal cancer was associated with lower mortality, with aHR of 0.824 (95%CI: 0.762–0.903) for overall survival and 0.856 (95%CI: 0.764–0.958) for cancer-specific survival (Table 2).

## 4. Discussion

In this retrospective population-based cohort study, we found that racial and subsite disparities in CRC outcomes differed between early-onset and late-onset cancer patients in Louisiana. Although racial disparities in cancer outcomes have been well-documented, numerous studies have shown that these disparities persist in CRC within the United States [1,2,4,13,15,16,17,18,19]. In the fully adjusted model, we found no significant racial disparities in either age group overall in Louisiana. After being stratified by tumor location, among early-onset patients, NHB with distal colon cancer had worse cancer-specific survival compared to NHW. Conversely, NHB with late-onset rectal cancer exhibited better overall and cancer-specific survival than NHW. Furthermore, survival differences by anatomic subsite varied by race and age group. EOCRC patients diagnosed with proximal colon cancer faced significantly higher risks of overall and cancer-specific survival compared to patients with distal colon cancer among NHW, which was not found among NHB. Additionally, among LOCRC patients, a subsite-specific survival disparity was found in NHB: those with rectal cancer had better overall and cancer-specific survival than those with distal colon cancer. This heterogeneity was not observed among NHW. To our knowledge, this is the first study to examine racial and subsite survival differences in colorectal cancer while considering the effect modification of both race and tumor location.

The etiology of anatomic subsite disparities in CRC survival is multifactorial and complex. Based on differences in embryologic origin and biological characteristics, the proximal and distal colon are often regarded as two distinct entities [30]. Additionally, proximal and distal colon tumors have significant differences in gene expression profiles [31]. For instance, microsatellite instability (MSI) positive tumors, particularly those classified as MSI-high (MSI-H), are more frequently located in the proximal colon and are generally associated with a better prognosis. In contrast, distal colon cancers are more likely to exhibit chromosomal instability (CIN), a feature linked to worse clinical outcomes [30,31,32,33]. Additionally, *KRAS* mutations are more common in proximal colon tumors [34]. Histological subtype may also contribute to location-specific disparities in CRC outcomes. Tumors with mucinous histology occur more commonly in the proximal colon compared with the distal colon, and these variants are typically associated with poorer survival [35]. Taken together, embryologic, molecular, and histopathologic factors highlight the biological heterogeneity between proximal and distal CRC and provide a partial explanation for the observed differences in patient outcomes across anatomic subsites.

Previous studies driven from Surveillance, Epidemiology, and End Results (SEER) data showed that NHB experience worse cancer-specific survival than NHW for both EOCRC and LOCRC after adjusting for relevant covariates [15,19]. In our analysis of Louisiana patients, we observed a similar association in unadjusted models for both EOCRC and LOCRC. However, after adjustment for sociodemographics, tumor characteristics, and treatment, significant racial disparities in overall or cancer-specific survival were identified only when stratified by tumor location. For example, NHB had a significantly higher hazard of cancer-specific death than NHW among those diagnosed with early-onset distal colon cancer. In contrast, among patients with late-onset rectal cancer, NHB had a lower risk of both all-cause and cancer-specific death.

While factors like access to care and lifestyle are associated with racial disparities in CRC mortality, numerous studies have suggested that racial disparities in CRC survival may, in part, be attributed to differences in tumor biology, particularly with respect to somatic mutation profiles. For example, somatic mutations in *KRAS*, *BRAF*, and *PIK3CA* have been shown to confer resistance to EGFR inhibition and are associated with increased mortality in CRC patients [36,37,38,39]. However, the prevalence of these oncogenic mutations varies by race. Patients of African ancestry exhibit a higher frequency of *KRAS* and *PIK3CA* mutations compared to White patients, while *BRAF* mutations are more commonly observed in White patients than in Black patients [34,37]. These patterns were observed in EOCRC patients, except for *PIK3CA* [40]. Hein et al. found that NHB and NHW with EOCRC had a similar prevalence of *PIK3CA* [40]. Additionally, among the MSI-H cohort, *PIK3CA* is more commonly altered in EOCRC patients [41]. Our findings of subsite-specific survival difference by race indicate that biological heterogeneity in tumor phenotypes may contribute to the observed disparities.

Numerous studies have reported that adult colorectal cancer patients with right-sided (proximal) colon cancer have worse survival outcomes compared to those with left-sided (distal) colon cancer [20,21,22,23]. In our study, similar findings were observed among NHW with EOCRC in Louisiana, for both overall and cancer-specific survival. However, among Louisiana patients with LOCRC, no significant differences in survival were found between proximal and distal colon cancers, which was consistent with other studies using SEER Medicare data [42]. Recent studies have also suggested that rectal cancer is associated with better survival compared to colon cancer [1]. In our analysis, among NHB with LOCRC, those with rectal cancer had slightly better survival than those with distal colon cancer. Conversely, among NHW with EOCRC, rectal cancer was associated with a higher risk of death for both overall and cancer-specific survival, although the difference was statistically significant only for cancer-specific survival. These findings suggest subsite-specific survival associations rather than inherent survival advantages and should be interpreted cautiously given the observational design.

Our findings underscore the need for heightened awareness among healthcare providers regarding EOCRC risk in NHB, especially for distal and rectal tumors, which are associated with worse outcomes. Colorectal cancer screening for average-risk adults beginning at age 45 years was formally incorporated into U.S. clinical guidelines following the U.S. Preventive Services Task Force (USPSTF) recommendation in May 2021 [43]. Although this updated guideline has the potential to improve early detection of CRC, healthcare providers must remain vigilant in identifying high-risk populations, particularly individuals with a family history of CRC and younger adult NHB, who experience an elevated risk. Recognizing these groups is essential to ensure timely evaluation of CRC-related symptoms, appropriate referral for diagnostic colonoscopy, and adherence to evidence-based treatment. In addition, EOCRC patients face significant challenges to quality of life due to the stage of life in which they are diagnosed, including financial and reproductive, and concern about long-term effects of treatment [44], some of which can be mitigated by early diagnosis.

From a public health perspective, targeted strategies are also needed to reduce structural barriers to screening among high-risk populations. These barriers include limited access to primary care, insurance coverage gaps, delays in follow-up after abnormal screening results, and insufficient education on healthy lifestyles, including balanced diets and regular physical activity. Although the present analyses focused on CRC survival, ongoing work will examine the distribution of environmental and other contributing factors associated with EOCRC. Given the limited understanding of EOCRC etiology, disaggregated analyses such as these are essential for generating context-specific insights that can inform precise public health interventions and clinical approaches in Louisiana.

This study has several notable strengths. First, it is a population-based study that uses high-quality data from a statewide cancer registry, with a very low percentage of patients who were lost to follow-up. In this dataset, only 3.5% of eligible CRC patients were lost to follow-up. Second, the LTR data include the underlying cause of death, obtained from either the state death file or the national death file, allowing us to assess racial and anatomic subsite differences in cancer-specific survival. Third, the significant geographic heterogeneity in EOCRC incidence and trends, including overall higher EOCRC incidence in the U.S. southern region, highlight the importance of state-based analyses such as these to identify appropriate regional public health strategies to address EOCRC [45]. Several limitations should be noted. First, we could not include other racial/ethnic groups beyond NHW and NHB in the CRC survival analyses by age group due to the small number of Louisiana cancer patients from other racial/ethnic groups. Second, using the census tract-level poverty as a proxy for individual socioeconomic status may have led to misclassification. Third, lack of detail regarding treatment regimens made it possible that differences in treatment may have contributed to differences in survival. Fourth, because of the lack of molecular tumor data in this retrospective cohort study, we were unable to examine the impact of differences in cancer molecular subtypes across races and tumor locations on CRC survival. Fifth, although known confounders were controlled for, residual unmeasured confounding may persist, including lifestyle factors such as diet and physical activity that are associated with improved CRC outcomes [46]. Well-designed observational studies can more precisely address the relationship between lifestyle factors and survival, which is especially important in EOCRC given the unique challenges of being diagnosed with cancer at a young age. Lastly, the generalizability of our findings may be limited by the restriction of the study population to colorectal cancer patients in Louisiana. Given the state’s unique demographic composition, with approximately one-third of the population identifying as Black, our results may not be fully representative of other states or the broader U.S. population.

## 5. Conclusions

Although EOCRC demonstrated better survival at the population level, this advantage was not uniform across anatomic subsites or racial groups. Our findings indicate that survival differences by race and tumor subsite vary between EOCRC and LOCRC. Racial disparities in survival emerged only after stratifying by tumor location, with distinct patterns observed between early- and late-onset patients. Tumor location is primarily associated with overall survival; however, once stratified by race, tumor location becomes associated with both survival outcomes, and these relationships differ across racial and age groups. In addition, improving early detection through appropriate screening for younger adults at high risk of CRC is crucial for identifying precancerous lesions and early-stage disease. Collectively, these results underscore the complexity of CRC survival disparities and support the need for subsite- and age-specific analytic frameworks. Future studies integrating molecular tumor characteristics are essential to elucidate the biological mechanisms underlying these observed associations.

## Figures and Tables

**Table 1:** Characteristics of colorectal cancer patients by early- and late-onset status, Louisiana, years 2011–2022.

Variables	N = 23,738	EOCRC (N = 2530)	LOCRC (N = 21,208)	*p*-Value
Sociodemographic Factors				
Race				<0.0001
Non-Hispanic White	15,880 (66.9)	1581 (62.49)	14,299 (67.42)	
Non-Hispanic Black	7858 (33.1)	949 (37.51)	6909 (32.58)	
Sex				<0.0001
Male	12,827 (54.0)	1301 (51.42)	11,526 (54.35)	
Female	10,911 (46.0)	1229 (48.58)	9682 (45.65)	
Marital status				0.4678
Married	11,491 (48.4)	1224 (48.38)	10,267 (48.41)	
Single	11,130 (46.9)	1199 (47.39)	9931 (46.83)	
Unknown	1117 (4.7)	107 (4.23)	1010 (4.76)	
Insurance				<0.0001
Private insurance	8752 (36.9)	1477 (58.38)	7275 (34.3)	
Medicare/other government	9730 (41.0)	170 (6.72)	9560 (45.08)	
Medicaid	3977 (16.8)	663 (26.21)	3314 (15.63)	
Uninsured/unknown	1279 (5.4)	220 (8.7)	1059 (4.99)	
Census tract level poverty				0.0262
<10%	4720 (19.9)	537 (21.23)	4183 (19.72)	
10%–<20%	9037 (38.1)	990 (39.13)	8047 (37.94)	
≥20%	9981 (42.0)	1003 (39.64)	8978 (42.33)	
Urban/Rural				0.0760
Urban	19,494 (82.1)	2110 (83.4)	17,384 (81.97)	
Rural	4244 (17.9)	420 (16.6)	3824 (18.03)	
Clinical Factors				
Site				<0.0001
Proximal colon	9794 (41.3)	651 (25.73)	9143 (43.11)	
Distal colon	6483 (27.3)	755 (29.84)	5728 (27.01)	
Rectum	7461 (31.4)	1124 (44.43)	6337 (29.88)	
SEER summary stage				<0.0001
Localized	9618 (40.5)	853 (33.72)	8765 (41.33)	
Regional	8793 (37.0)	997 (39.41)	7796 (36.76)	
Distant	5327 (22.4)	680 (26.88)	4647 (21.91)	
Grade				0.4194
Low	17,152 (72.3)	1837 (72.61)	15,315 (72.21)	
High	3560 (15.0)	359 (14.19)	3201 (15.09)	
Unknown	3026 (12.7)	334 (13.2)	2692 (12.69)	
Tumor number				<0.0001
Single primary site	17,538 (73.9)	2244 (88.7)	15,294 (72.11)	
Multiple primary sites	6200 (26.1)	286 (11.3)	5914 (27.89)	
Comorbidity				<0.0001
None ^#^	16,070 (67.7)	2131 (84.23)	13,939 (65.73)	
CCI score = 1	4718 (19.9)	302 (11.94)	4416 (20.82)	
CCI score ≥ 2	2950 (12.4)	97 (3.83)	2853 (13.45)	
Treatment				
Surgery				0.2735
No	3906 (16.5)	397 (15.69)	3509 (16.55)	
Yes	19,832 (83.5)	2133 (84.31)	17,699 (83.45)	
Chemotherapy				<0.0001
No	12,713 (53.6)	876 (34.62)	11,837 (55.81)	
Yes	10,019 (42.2)	1565 (61.86)	8454 (39.86)	
Unknown	1006 (4.2)	89 (3.52)	917 (4.32)	
Radiation				<0.0001
No	19,884 (83.8)	1902 (75.18)	17,982 (84.79)	
Yes	3549 (15.0)	595 (23.52)	2954 (13.93)	
Unknown	305 (1.3)	33 (1.3)	272 (1.28)	
All-cause death				<0.0001
Alive	11,976 (50.5)	1652 (65.3)	10,324 (48.68)	
Death	11,762 (49.5)	878 (34.7)	10,884 (51.32)	
5-year survival rate% (95%CI)	54.0 (53.4–54.7)	65.1 (63.0–67.1)	52.7 (52.0–53.5)	<0.0001
Cancer-specific death				0.0281
Alive or died in other cause	16,160 (68.1)	1771 (70)	14,389 (67.85)	
Died in cancer related cause	7578 (31.9)	759 (30)	6819 (32.15)	
5-year survival rate% (95%CI)	65.8 (65.1–68.1)	68.5 (66.4–70.4)	65.5 (64.7–66.2)	<0.0001

Abbreviations: EOCRC, early-onset colorectal cancer; LOCRC, late-onset colorectal cancer; CCI, Charlson comorbidity index; CI, confidence interval. ^#^ No comorbidity documented in the medical chart.

**Table 2:** Hazard ratio (HR) and 95% confidence interval (95%CI) of race, site, and stage on overall and cancer-specific survival by age group.

Model	Overall Survival		Cancer-Specific Survival	
	EOCRC HR (95%CI)	LOCRC HR (95%CI)	EOCRC HR (95%CI)	LOCRC HR (95%CI)
Race: NHW as reference				
Model 1—NHB	**1.307 (1.143–1.494)**	1.036 (0.995–1.078)	**1.269 (1.099–1.466)**	**1.127 (1.072–1.184)**
Model 2—NHB	**1.172 (1.006–1.367)**	0.987 (0.944–1.032)	1.175 (0.996–1.385)	1.007 (0.953–1.064)
Model 3—NHB	1.110 (0.951–1.295)	0.974 (0.931–1.018)	1.101 (0.932–1.300)	0.993 (0.939–1.050)
Model 4: Model 3 + race × site interaction				
Proximal: NHB	0.943 (0.730–1.218)	0.980 (0.921–1.044)	0.946 (0.714–1.253)	1.070 (0.989–1.158)
Distal: NHB	1.254 (0.962–1.633)	1.045 (0.966–1.131)	**1.358 (1.024–1.801)**	1.015 (0.918–1.122)
Rectal: NHB	1.155 (0.920–1.451)	**0.899 (0.831–0.973)**	1.061 (0.829–1.357)	**0.873 (0.793–0.960)**
Subsite: Distal colon as reference				
Model 1—Proximal	**1.297 (1.089–1.545)**	**1.133 (1.082–1.186)**	**1.221 (1.011–1.476)**	**1.082 (1.020–1.148)**
Model 1—Rectal	1.028 (0.875–1.208)	0.966 (0.918–1.016)	1.034 (0.871–1.227)	**1.071 (1.005–1.140)**
Model 2—Proximal	**1.215 (1.018–1.451)**	1.012 (0.965–1.061)	1.155 (0.953–1.400)	1.056 (0.995–1.122)
Model 2—Rectal	**1.190 (1.007–1.407)**	1.023 (0.972–1.077)	**1.213 (1.014–1.450)**	**1.116 (1.047–1.189)**
Model 3—Proximal	**1.225 (1.026–1.462)**	0.999 (0.953–1.047)	1.162 (0.959–1.408)	1.035 (0.974–1.099)
Model 3—Rectal	1.176 (0.972–1.421)	0.910 (0.859–0.964)	1.184 (0.965–1.453)	0.943 (0.878–1.014)
Model 4: Model 3 + race × site interaction				
NHW: Proximal	**1.407 (1.102–1.796)**	1.022 (0.964–1.082)	**1.379 (1.057–1.799)**	1.015 (0.941–1.094)
NHW: Rectal	1.221 (0.967–1.543)	0.958 (0.895–1.027)	**1.314 (1.022–1.690)**	0.995 (0.914–1.084)
NHB: Proximal	1.059 (0.820–1.367)	0.958 (0.885–1.037)	0.960 (0.729–1.264)	1.069 (0.969–1.180)
NHB: Rectal	1.126 (0.855–1.482)	**0.824 (0.752–0.903)**	1.026 (0.763–1.381)	**0.856 (0.764–0.958)**

Abbreviations: EOCRC, early-onset colorectal cancer; LOCRC, late-onset colorectal cancer; NHW, non-Hispanic Whites; NHB, non-Hispanic Blacks. HR, hazard ratio; CI, confidence interval. Model 1: Unadjusted model. Model 2: Adjusted for sociodemographics including age at cancer diagnosis, sex, marital status, insurance, census tract level poverty, and urban/rural status; tumor characteristics including tumor site, cancer stage, grade, and number of tumors; and comorbidity. Model 3: Adjusted for sociodemographics, tumor characteristics, comorbidity, and treatment including surgery, chemotherapy, and radiation. **Bold estimates** indicate statistical significance. A statistically significant race-by-subsite interaction indicated that the association between race and survival varied by tumor location. For example, among early-onset distal colon cancer patients, non-Hispanic Black individuals had a 35.8% higher risk of overall mortality compared with non-Hispanic White individuals (adjusted HR = 1.358; 95%CI: 1.024–1.801).

## Data Availability

The data that supports the findings of this study are available on request from the corresponding author. The data is not publicly available due to privacy or ethical restrictions.
